# Structural insights of non-canonical U•U pair and Hoogsteen interaction probed with Se atom

**DOI:** 10.1093/nar/gkt799

**Published:** 2013-09-05

**Authors:** Jia Sheng, Jianhua Gan, Alexei S. Soares, Jozef Salon, Zhen Huang

**Affiliations:** ^1^Department of Chemistry, Georgia State University, Atlanta, GA, 30303, USA and ^2^Department of Biology, Brookhaven National Laboratory, Upton, NY, 11973, USA

## Abstract

Unlike DNA, in addition to the 2′-OH group, uracil nucleobase and its modifications play essential roles in structure and function diversities of non-coding RNAs. Non-canonical U•U base pair is ubiquitous in non-coding RNAs, which are highly diversified. However, it is not completely clear how uracil plays the diversifing roles. To investigate and compare the uracil in U-A and U•U base pairs, we have decided to probe them with a selenium atom by synthesizing the novel 4-Se-uridine (^Se^U) phosphoramidite and Se-nucleobase-modified RNAs (^Se^U-RNAs), where the exo-4-oxygen of uracil is replaced by selenium. Our crystal structure studies of U-A and U•U pairs reveal that the native and Se-derivatized structures are virtually identical, and both U-A and U•U pairs can accommodate large Se atoms. Our thermostability and crystal structure studies indicate that the weakened H-bonding in U-A pair may be compensated by the base stacking, and that the stacking of the *trans*-Hoogsteen U•U pairs may stabilize RNA duplex and its junction. Our result confirms that the hydrogen bond (O4^…^H-C5) of the Hoogsteen pair is weak. Using the Se atom probe, our Se-functionalization studies reveal more insights into the U•U interaction and U-participation in structure and function diversification of nucleic acids.

## INTRODUCTION

Unlike natural DNA, which merely stores genetic information in cells (1), natural RNA is highly diversified in structure and function. Because of the RNA diversity, RNA plays essential functions in cells and expands complexity of living systems by serving as genetic information carrier, catalyst and regulator (2–10). Recently, tremendous functional RNAs have been discovered as non-coding RNAs (ncRNA), such as ribozymes, riboswitches, small interfering RNA (siRNA), microRNA (miRNA), small nuclear RNA (snRNA) and RNAs regulating biological pathways. ncRNAs can control gene expressions selectively through transcription and translation regulations (11,12), participate in chromatin silencing and remodeling (13), regulate the retroviruses activity (14), catalyze biochemical reactions (15,16), recognize metabolites (17), as well as facilitate gene function study and drug discovery (18,19). ncRNAs play highly specific roles by folding into various 3D structures and binding specifically with other molecules or ligands (such as proteins and metabolites), which may trigger cascades of biological events.

However, considering the similar chemical structures of nuclei acid building blocks (such as almost the same nucleobases in RNA and DNA), it is striking that RNA with the extra 2′-OH is able to establish much more diversified structures and functions than DNA (20,21). In addition to the 2′-OH group, it appears that the RNA modifications and non-canonical base pairings are the two major strategies to overcome the structural homogeneity limit caused by the four similar nucleobases and to achieve huge diversities in both structure and function (22–24). Especially, uracil nucleobase can form multiple non-canonical base pairings and play essential roles in diversifying RNA structure and function. Non-canonical U•U base pair is ubiquitous in ncRNA, and Watson–Crick U-A pair can often be replaced with U-G wobble pair without significant duplex destablization, which increases structure and function diversity of ncRNAs. U•U pairs are often observed in RNA duplex joinction and loops (25–27), whereas U-A pair is normally not formed at these places. Replacing U-A pair in duplex with U•U pair significantly destablizes the duplex structure. It is not completely clear how uracil plays the diversifying roles in these base pairs to achieve the structure and function diversity. To investigate and compare the uracil roles played in these non-canonical and canonical pairs, we have decided to probe the U•U and U-A pairs with a Se atom, where the exo-4-oxygen of uracil is replaced by selenium.

Though 4-Se-uridine was synthesized over three decades ago (28,29), it has not been incorporated into RNAs because of the synthetic challenges. Recently, our successes on the synthesis and biophysical studies of the Se-nucleobase modifications (30–35) have encouraged us to overcome the ^Se^U-RNA synthesis challenge, meet the urgent needs in ncRNA investigation and probe U-A and U•U pairs by a Se atom. Herein, we report the first synthesis of the 4-Se-uridine phosphoramidite (^Se^U) and the corresponding ^Se^U-RNAs by replacing 4-oxygen with selenium. We have found that this Se-modification does not cause significant perturbation and that the native and modified structures are virtually identical. We also found that via the stacking and hydrogen bonding, the uracil nucleobase interacts differently in RNA duplex and duplex junction. Moreover, the accommodation of the larger selenium atom by both U-A and U•U pairs implies the RNA flexibility. Our studies suggest that by presenting their different faces and edges, uracil and uridine are capable of diversifing structure and function of ncRNAs. Furthermore, this Se-modified uridine offers the Se-RNAs with additional UV absorption (λ_max_: 370 nm; ε: 1.30 × 10^4^ M^−^^1^cm^−^^1^). Excitingly, after a single-oxygen atom replacement with selenium, we have observed for the first time the color RNAs (light yellow) as well as color RNA crystals (dark yellow). The color property of the ^Se^U-RNAs is unique and has great potentials in RNA visualization, detection, spectroscopic study and crystallography of RNAs and protein-RNA complexes and interactions, demonstrating the usefulness of selenium-derivatized nucleic acids (SeNA) (36,37) in structural biology. In addition, both the anomalous phasing and molecular replacement approaches result in the identical crystal structures. Our new method provides a unique atomic tool for probing structure and function of ncRNAs and their protein complexes.

## MATERIALS AND METHODS

### Synthesis of the 4-Se-uridine phosphoramidite

#### 3-(1-((2R,3S,4S,5R)-5-((bis(4-methoxyphenyl)(phenyl)methoxy)methyl)-3-(tert-butyldimethyl-silyloxy)-4-hydroxy-tetrahydrofuran-2-yl)-2-oxo-1,2-dihydropyrimidin-4-ylselanyl)propanenitrile

To a dry THF solution (10 ml) of the starting material compound (1, 1.34 g, 2 mmol), 4,4′-dimethylamino-pyridine (24.5 mg, 0.2 mmol) and triethylamine (0.56 ml, 4 mmol) under argon, the dry tetrahydrofuran (THF) solution (10 ml) of 2,4,6-trisopropylbenzenessulfonyl chloride (906 mg, 3.0 mmol) was added dropwisely. The reaction was stirred for 1 h before it is finished (monitor by thin layer chromatography (TLC), 5% methanol in dichloromethane). At the same time, the NaBH_4_ suspension (250 mg of NaBH_4_ in 3 ml of EtOH) was injected into a flask containing di(2-cyanoethyl) diselenide [(NCCH_2_CH_2_Se)_2_, 0.3 ml, d = 1.8 g/ml, 2.0 mmol] and THF (10 ml) in an ice bath with argon. The yellow color of the diselenide disappeared in ∼15 min, giving an almost colorless suspension of sodium selenide (NCCH_2_CH_2_SeNa). Then, the reacted solution of compound **1** was slowly injected into this selenide solution. After the selenium incorporation was completed in 45 min (monitored on TLC, 5% MeOH in CH_2_Cl_2_, product R*_f_* = 0.60), water (100 ml) was added to the reaction flask. The solution was adjusted to pH 7–8 using CH_3_COOH (10%) and was then extracted with ethyl acetate (3 × 100 ml). The organic phases were combined, washed with NaCl (sat., 100 ml), dried over MgSO_4_ (s) for 30 min and evaporated to minimum volume under reduced pressure. The crude product was then dissolved in methylene chloride (5 ml) and purified on a silica gel column equilibrated with hexanes/methylene chloride (1:1). The column was eluded with a gradient of methylene chloride (CH_2_Cl_2_, 0.5%, 1% and 2% MeOH in CH_2_Cl_2_, 300 ml each). After the collected fraction evaporation and dry under high vacuum, pure compound **2** was obtained as a slightly yellow foam product (1.27 g, 81% yield). ^1^H-NMR (400 MHz, CDCl_3_) δ: 0.21 (s, 3H, CH_3_), 0.38 (s, 3H, CH_3_), 0.95 (s, 6H, 2× CH_3_), 2.31-2.37 (m, 1H, H-2′), 3.00 (dd, *J* = 6.5 and 6.7 Hz, 2H, CH_2_-Se), 3.37-3.41 (m, 2H, CH_2_-CN), 3.50-3.52 (m, 2H, 1H-5′), 3.81 (s, 6H, 2× OCH_3_), 4.17–4.22 (m, 1H, H-3′), 4.31 (s, 1H, 3′-OH), 4.40–4.50 (m, 1H, H-4′), 5.78 (s, 1H, H-1′), 5.90 (d, 1H, *J* = 6.8 Hz, H-5), 6.8–6.90 (m, 4H, aromatic), 7.20–7.46 (m, 9H, aromatic), 8.31 (d, 1H, *J* = 6.8 Hz, H-6). ^13^C-NMR (100 MHz, CDCl_3_) δ: −4.30, −4.40 (CH_3_), 18.1 (CH_2_-CN), 19.0 (CH_2_-CH_2_-CN), 20.5 [(CH_3_)_2_C(t-Bu)], 25.9 (CH_3_), 55.3 (OCH_3_), 68.7 (C-3′), 76.4 (C-2′), 83.1 (C-4′), 91.0 (C-1′), 106.0 (C-5), 118.8 (CN), 113.3, 127.1, 128.0, 128.2, 130.1, 135.0, 135.3, 144.2, 158.7 (Ar-C), 140.4 (C-6), 153.3 (C-2), 175.0 (C-4). HRMS (ESI-TOF): molecular formula, C_39_H_49_N_3_O_7_SeSi; [M+H]^+^: 778.2413 (calc.778.2426).

#### (2R,3S,4S,5R)-2-((bis(4-methoxyphenyl)(phenyl)methoxy)methyl)-4-(tert-butyldimethylsilyloxy)-5-(4-(2-cyanoethylselanyl)-2-oxopyrimidin-1(2H)-yl)-tetrahydrofuran-3-yl-2-cyanoethyl diisopropylphosphoramidite

To the flask (25 ml) containing **2** (453 mg, 0.68 mmol) under argon, dry methylene chloride (2.5 ml), *N*,*N*-diisopropylethylamine (0.17 ml, 1.03 mmol, 1.5 eq.), and 2-cyanoethyl *N*,*N*-diisopropyl-chlorophosphoramidite (195 mg, 0.83 mmol, 1.2 eq.) were added sequentially (3). The reaction mixture was stirred at −10°C in an ice-salt bath under argon for 10 min, followed by removal of the bath. The reaction was completed in 2 h at room temperature, generating a mixture of two diastereomers (indicated by TLC, 5% MeOH in CH_2_Cl_2_, product R*_f_* = 0.63 and 0.68). The reaction was then quenched with NaHCO_3_ (5 ml, sat.) and stirred for 5 min, followed by the extraction with CH_2_Cl_2_ (3 × 8 ml). The combined organic layer was washed with NaCl (10 ml, sat.) and dried over MgSO_4_ (s) for 30 min, followed by filtration. The solvent was then evaporated under reduced pressure, and the crude product was re-dissolved in CH_2_Cl_2_ (2 ml). This solution was added drop-wise to cold petroleum ether (or hexane) (200 ml) under vigorous stirring, generating a white precipitate. The petroleum ether layer was decanted. The crude product was re-dissolved again in CH_2_Cl_2_ (2 ml) and then loaded on Al_2_O_3_ column (neutral) that was equilibrated with CH_2_Cl_2_/Hexanes (1:1). The column was eluded with a gradient of methylene chloride and ethyl acetate [CH_2_Cl_2_ to CH_2_Cl_2_/EtOAc (7:3)]. After solvent evaporation and dry over high vacuum, the compound **3** (612 mg) was obtained as a white foamy product (92% yield). ^1^H-NMR (400 MHz, CDCl_3_, two sets of signals from a mixture of two diastereomers) δ: 0.2–0.4 (m,12H, 4 × CH_3_), 0.85–1.20 [m, 36H, 8 × CH_3_-ipr and 4× Si(CH_3_)], 2.30–2.38 and 2.70–2.82 (2× m, 4H, 2× H-2′), 2.34 and 2.64 (2× t, *J* = 6.4 Hz, 4H, 2× O-CH_2_-CH_2_-CN), 3.00–3.04 (m, 4H, 2× Se-CH_2_-CH_2_-CN), 3.32–3.44 (m, 6H, 2× H-5′, 2× Se-CH_2_), 3.52–3.64 (m, 8H, 4× CH-ipr, 2× O-CH_2_-CH_2_-CN), 3.73–3.84 (m, 2H, 2× H-5′), 3.82 and 3.83 (2× s, 12H, 4× OCH_3_), 4.12–4.35 (m, 2H, 2× H-3′), 4.43–4.48 (m, 2H, 2× H-4′), 5.70–5.90 (m, 4H, 2× H-5 and 2× H-1′), 6.83–6.88 (m, 8H, aromatic), 7.27–7.43 (m, 18H, aromatic), 8.30 and 8.39 (2× s, 2H, 2× H-6). HRMS (ESI-TOF): molecular formula, C_48_H_64_N_5_O_8_PSeSi; [M + H]^+^: 978.3479 (calc. 978.3505).

#### Synthesis of the ^Se^U-RNAs

All the RNA oligonucleotides were chemically synthesized in 1.0 μmol scale on solid phase. The ultra-mild RNA phosphoramidites protected with 2′-TBDMS were used (Glen Research). The concentration of the ^Se^U-phosphoramidite was 0.08 M in acetonitrile, compared with the regular ones (0.1 M). Coupling was carried out using 5-(benzylmercapto)-1H-tetrazole solution (0.25 M) in acetonitrile with 12 min coupling time for both native and Se-modified phosphoramidites. Three percent trichloroacetic acid in methylene chloride was used for the 5′-detritylation. Synthesis was performed on control-pore glass (CPG-500) immobilized with the appropriate nucleoside through a succinate linker. All oligonucleotides were prepared in dimethoxy trityl (DMTr)-on form. After synthesis, the RNAs were cleaved from the solid support and fully deprotected by 0.05 M K_2_CO_3_ (methanol solution) for 8 h at room temperature, followed by neutralization, evaporation and the treatment of tetrabutylammonium fluoride (TBAF) solution (1 M in THF) for overnight. After desalting and HPLC purification, the 5′-DMTr group was removed by 3% aqueous solution of trichloroacetic acid, and the solution was neutralized to pH 7.0 with a freshly made triethylammonium acetate (TEAAc) buffer and precipitated with NaCl (final concentration: 0.3 M before ethanol addition) and ethanol (3 volumes). The ethanol suspension was placed at −80^°^C for 1 h, followed by centrifugation to collect the RNAs.

#### HPLC analysis and purification

The RNA oligonucleotides were analyzed and purified by reverse-phase high performance liquid chromatography (RP-HPLC) in DMTr-on form. After the TBAF desilylation and desalting with sephadex G-25, HPLC purification was carried out using a 21.2 × 250 mm Zorbax, RX-C8 column at a flow rate of 6 ml/min. Buffer A consisted of 10 mM TEAAc (pH 7.1), whereas buffer B contained 50% acetonitrile and 10 mM TEAAc (pH 7.1). Similarly, the HPLC analysis was performed on a Zorbax SB-C18 column (4.6 × 250 mm) at a flow of 1.0 ml/min using the same buffer system. The DMTr-on oligonucleotides were eluded in a 20-min linear gradient of 100% buffer A to 100% buffer B. The HPLC analysis for both DMTr-on and DMTr-off oligonucleotides were carried out with up to 60% of buffer B in a linear gradient in the same period of time. The collected fractions were lyophilized, and the purified RNAs were re-dissolved in water for the detritylation and precipitation steps.

#### Thermodenaturation of the ^Se^U-RNAs

Solutions of the duplex RNAs (1 or 2 μM) were prepared by dissolving the purified RNAs in sodium phosphate [10 mM (pH 6.5)] buffer containing 100 mM NaCl. The solutions were heated to 75°C for 3 min, then cooled down slowly to room temperature and stored at 4°C overnight before Tm measurement. Before thermal denaturation, the Se-RNA samples were bubbled with argon for 5 min. Each denaturizing curves were acquired at 260 nm by heating and cooling from 5 to 70°C for four times in a rate of 0.5°C/min, using Cary-300 UV-Visible spectrometer equipped with temperature controller system.

#### Se-RNA crystallization and diffraction data collection

The purified RNA oligonucleotides (1 mM) were heated to 70°C for 2 min and cooled down slowly to room temperature. Both native buffer and Nucleic Acid Mini Screen Kit (Hampton Research) were applied to screen the crystallization conditions at different temperatures using the hanging drop method by vapor diffusion (1 µl of RNA and 1 µl of buffer). Thirty percent glycerol, PEG 400 or the perfluoropolyether was used as a cryoprotectant during the crystal smounting, and data collection was taken under the liquid nitrogen stream at 99°K. The Se-RNA crystal data were collected at beam line X12B and X12C in NSLS, Brookhaven National Laboratory. A number of crystals were screened to find the ones with strong anomalous scattering at the K-edge absorption of selenium. The distance of the detector to the crystals was set to 150 mm. The radiation wavelength at 0.9795 Å was chosen for diffraction data collection and selenium single-wavelength anomalous dispersion (SAD) phasing. The crystals were exposed for 10 s per image with 1° oscillation, and a total of 180 images were taken for each data set. All data were processed using HKL2000 and DENZO/SCALEPACK (38).

#### Structure determination and refinement

The structures of Se-RNAs were solved by both SAD with HKL2MAP and molecular replacement with Phaser (39), followed by the refinement with Refmac. Both SAD phasing and molecular replacement led to the same crystal structure. The refinement protocol includes simulated annealing, positional refinement, restrained B-factor refinement and bulk solvent correction. The stereo-chemical topology and geometrical restrain parameters of DNA/RNA (40) have been applied. The topologies and parameters for the uridine modified with selenium (US) were constructed and applied. After several cycles of refinement, a number of highly ordered waters were added. Finally, the occupancies of selenium were adjusted. Cross-validation (41) with a 5–10% test set was monitored during the refinement. The σA-weighted maps (42) of the (2 m|Fo| - D|Fc|) and the difference (m|Fo| - D|Fc|) density maps were computed and used throughout the model building.

## RESULTS AND DISCUSSION

### Synthesis of the 4-Se-uridine (^Se^U) phosphoramidite

**Scheme 1.**
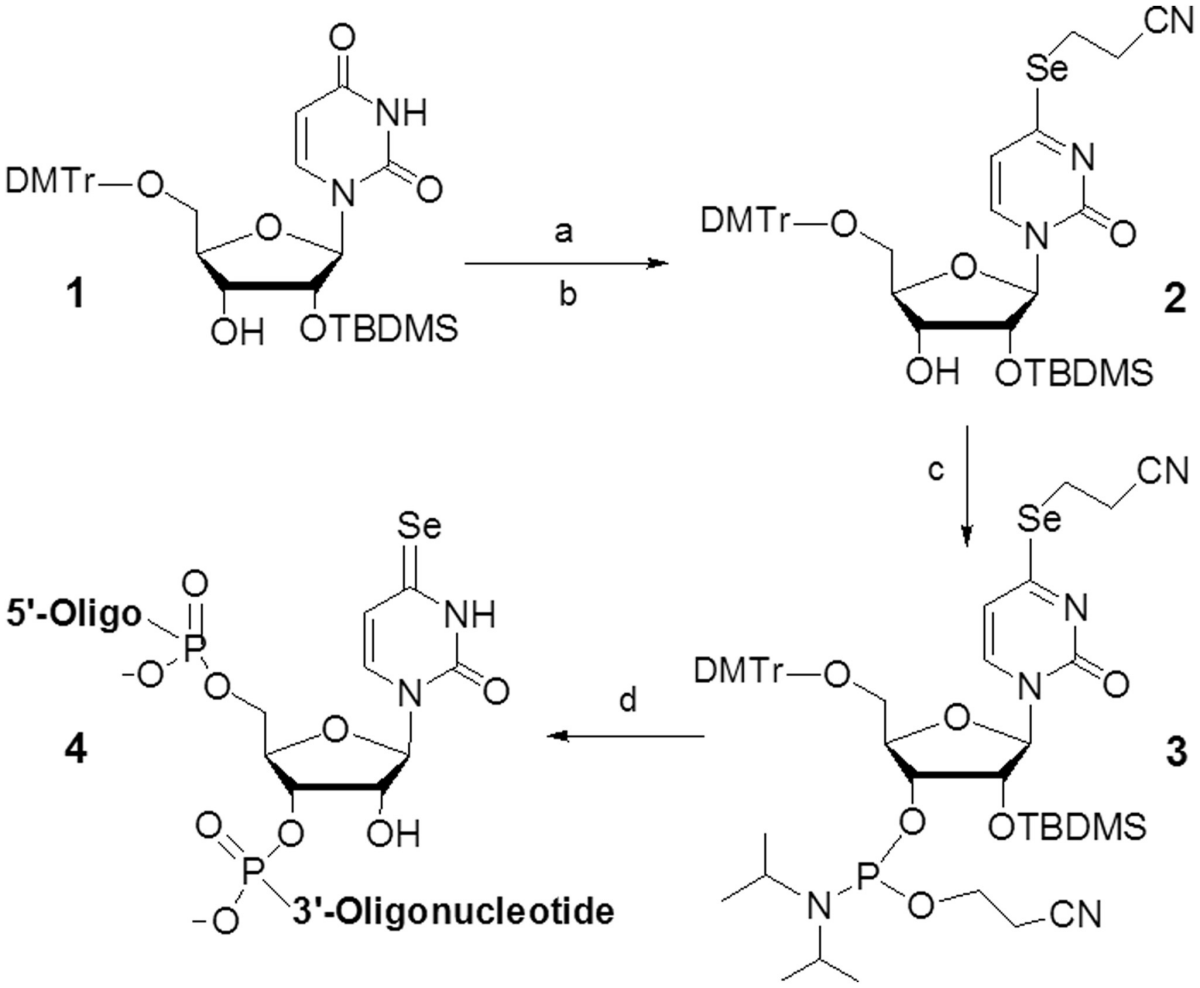
Synthesis of ^Se^U-phosphoramidite (**3**) and ^Se^U-RNAs (**4**). Reagents and conditions: (**a**) TIBS-Cl, 4,4′-dimethylamino-pyridine, CH_2_Cl_2_, room temperature; (**b**) (NCCH_2_CH_2_Se)_2_/NaBH_4_, EtOH; (**c**) 2-cyanoethyl N,N-diisopropylchloro-phosphoramidite and N,N-diisopropylethylamine in CH_2_Cl_2_; (**d**) the solid-phase synthesis. TIBS-Cl: 2,4,6-(triisopropylbenzene)sulfonyl chloride.

We have developed a facile strategy to synthesize the Se-phosphoramidite. As showed in Scheme 1, our synthesis started from the partially protected 2′-TBDMS-5′-trityl-uridine (1). To simplify the synthesis, we used a bulky reagent (2,4,6-triisopropylbenzenesulfonyl chloride, TIBS-Cl) to selectively activate position 4, thus avoiding the protection and deprotection steps of the 3′-hydroxyl group. Without purifying the activated intermediate, the selenium functionality was introduced by substituting TIBS group at position 4 with 2-cyanoethylselenide in the yield of 81%. Sodium 2-cyanoethylselenide was generated by the reduction of di-(2-cyanoethyl) diselenide with NaBH_4_ in ethanol solution (30). This protected Se-functionality is compatible with the solid-phase synthesis and can be removed by weak base treatment (K_2_CO_3_ in methanol). Finally, the 4-Se-uridine derivative (**2**) was converted to the corresponding phosphoramidite (**3**) in 92% yield. The analysis data are shown in the supporting information (Supplementary Figures S1–S7).

#### Synthesis of the SeU-RNAs

**Figure 1.**
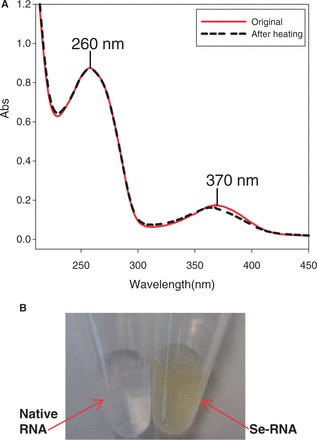
The UV spectra and color of the ^Se^U-RNA. (**A**) red line (λ_max_ = 260 and 370 nm): UV spectrum of the ^Se^U-RNA (5′-G-^Se^U-GUACAC-3′) without heating; black broken line (λ_max_ = 260 and 367 nm): UV spectrum of the ^Se^U-RNA after heating at 70°C for 8 h; (**B**) the ^Se^U-RNA (yellow, 1.0 mM) and the corresponding native RNA (colorless, 1.0 mM).

**Figure 2.**
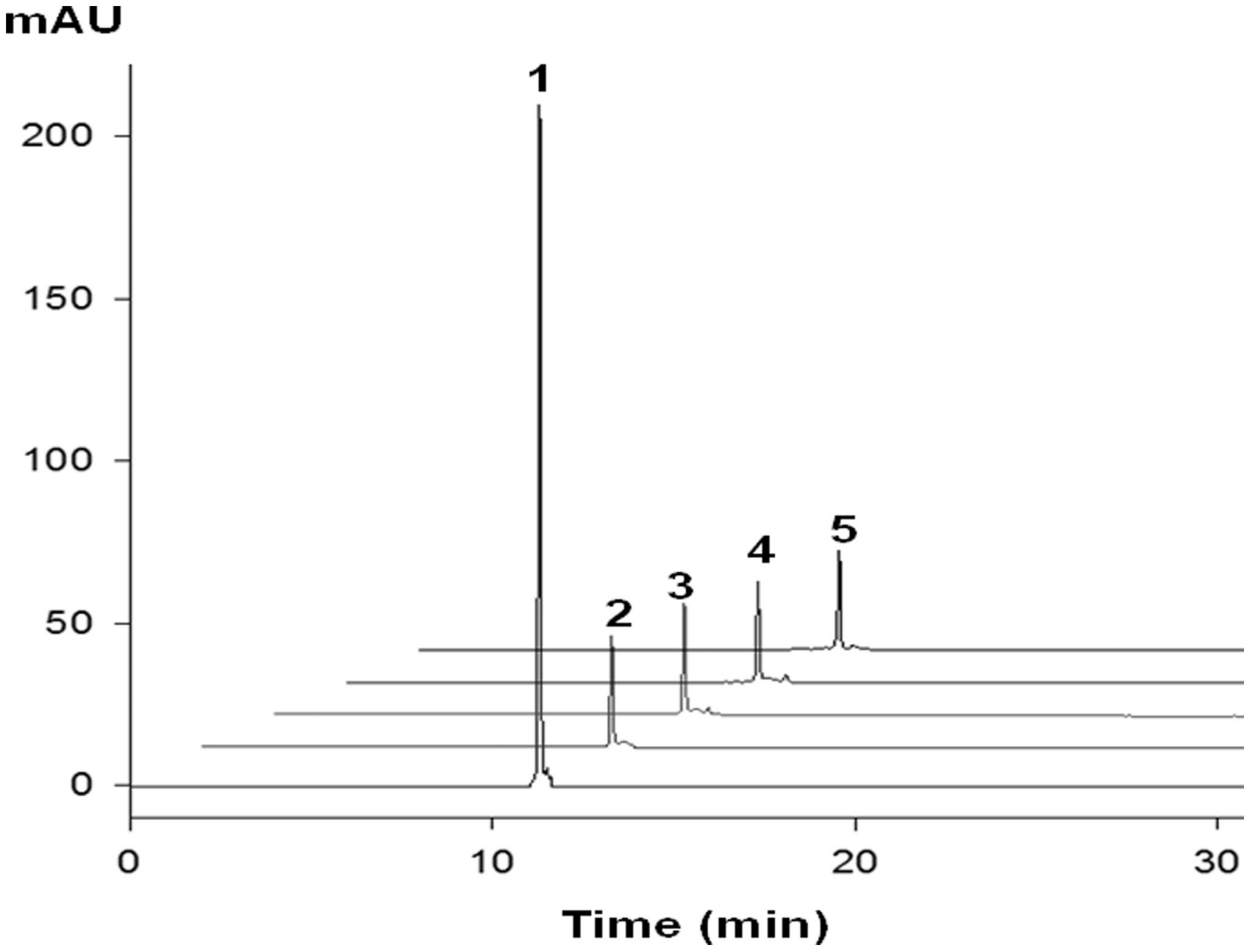
Thermal stability analysis of ^Se^U-RNA (5′-G-^Se^U-GUACAC-3′). HPLC profile 1 and 2 (without heating of the Se-RNA) monitored at 260 and 370 nm, respectively. HPLC profile 3, 4 and 5 (monitored at 370 nm) were analysis of the Se-RNA heated at 70°C for 2, 5 and 8 h, respectively.

The ultramild phosphoramidites, where the base-labile protecting groups can be deprotected with a weak base (K_2_CO_3_ in methanol) (30,32,33,35,43), were used because the 4-Se-functionality is sensitive to strong base cleavage (such as ammonia, causing deselenization). We found that this Se-modified phosphoramidite is compatible with the longer coupling time (12 min), I_2_ oxidation and trichloroacetic acid treatment without deselenization. In the case of RNAs containing multiple guanosine residues, phenoxyacetic anhydride (Pac_2_O) instead of acetic anhydride was used in the capping step to avoid the acetylation of guanosine, which is difficult to remove under the mild deprotecting conditions (K_2_CO_3_ in methanol). All Se-RNAs were synthesized in DMTr-on form, followed by cleavage and deprotection with 0.05 M methanol solution of K_2_CO_3_. After the deprotection, the solution was carefully neutralized with 1 M HCl and evaporated to dryness. Then the 2′-TBDMS groups were removed by treating with 1 M TBAF solution in THF at room temperature overnight. After desilylation and desalting, a typical HPLC profile of the crude Se-RNAs is shown in Supplementary Figure S8, which indicates a high coupling yield of the Se-uridine phosphoramidite (96%), compared with incorporation of the non-modified phosphoramidites. After desalting with Sephadex-G25 matrix, the pure Se-RNAs were obtained by RP-HPLC purification, followed by the mild detritylation (44). Several ^Se^U-RNAs containing Watson–Crick U-A and Hoogsteen U•U pairs were synthesized, purified and characterized (Table 1 and Supplementary Figures S8 and S9). Excitingly, we observed for the first time that the RNA with the single Se-atom substitution is visible and has yellow color. UV-vis spectroscopic study indicated the Se-RNA with λ_max_ at 260 and 370 nm (Figure 1) resulted from the native nucleobases and ^Se^U, respectively. The color RNAs can be used as potential probes for many biochemical and biomedical applications. We also found that the Se-RNA crystals are yellow color, indicating this Se-derivatization is especially useful for the crystallization screening of RNAs and protein-RNA complexes. The color is due to the ease of the electron delocalization on the nucleobase after the selenium derivatization, thereby red-shifting the spectrum significantly by over 100 nm. Furthermore, it is worth mentioning that this Se-functionality is relatively stable. After heating the Se-RNA at 70^°^C for 8 h, no significant decomposition was observed, indicated by UV and HPLC analyses (Figures 1A and 2).

**Table 1.** MALDI-TOF-MS Analysis of ^Se^U-RNA

Entry	Se-RNAs	Measured (calcd) m/z
1	5′-U-^Se^U-CGCG-3′ (C_56_H_71_N_20_O_41_P_5_Se)	[M + H]^+^: 1915.4 (1915.2)
2	5′-G-^Se^U-GUACAC-3′ (C_76_H_95_N_30_O_53_P_7_Se)	[M + H]^+^: 2573.3 (2573.3)
3	5′-GUG-^Se^U-ACAC-3′ (C_76_H_95_N_30_O_53_P_7_Se)	[M + H]^+^: 2573.5 (2573.3)
4	5′-AUGG-^Se^U-GCUC-3′ (C_85_H_106_N_32_O_62_P_8_Se)	[M + H]^+^: 2895.3 (2895.7)
5	5′-CGCGAA-^Se^U-UCGCG-3′ (C_114_H_144_N_46_O_81_P_11_Se)	[M + H]^+^: 3873.3 (3874.5)
6	5′-CGCGAAU-^Se^U-CGCG-3′ (C_114_H_144_N_46_O_81_P_11_Se)	[M + H]^+^: 3874.0 (3874.3)
7	5′-U-^Se^U-AUAUAUAUAUAA-3′ (C_133_H_162_N_49_O_95_P_13_Se)	[M + H]^+^: 4449.7 (4449.6)
8	5′-AA-^Se^U-A(2′-SeMe-U)AUAUAUAUU-3′ (C_134_H_164_N_49_O_94_P_13_Se_2_)	[M + H]^+^: 4526.4 (4526.4)
9	5′-GG-^Se^U-AUUGCGGUACC-3′ (C_133_H_165_N_52_O_97_P_13_Se)	[M + H]^+^: 4526.4 (4526.7)
10	5′-A-^Se^U-CACCUCCUUA-3′ (C_111_H_141_N_38_O_82_P_11_Se)	[M + H]^+^: 3740.8 (3740.2)
11	U-^Se^U-AGCUAGCU (C_94_H_117_N_34_O_69_P_9_Se)	[M + H]^+^: 3186.2 (3185.9)
12	U-^Se^U-CGCGAUCGCG (C_113_H_142_N_43_O_83_P_11_Se)	[M + H]^+^: 3851.7 (3851.3)
13	U-^Se^U-CAUGUGACC (C_103_H_129_N_37_O_76_P_10_Se)	[M + H]^+^: 3489.8 (3490.4)

### Determination of extinction coefficient of ^Se^U (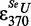)

**Figure 3.**
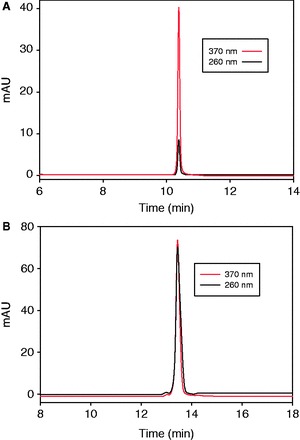
Calculation of 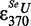 and 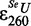 via RP-HPLC analysis. (**A**) HPLC profile of 3′-^Se^UMP at 260 nm (solid line) and 370 nm (dash line). (**B**) HPLC profile of 5′-^Se^UU-3′ dimer at 260 nm (solid line) and 370 nm (dash line). The samples (3′-^Se^UMP and 5′-^Se^UU-3′) were analyzed on a Welchrom XB-C18 column (4.6 × 250 mm, 5 μ) at a ﬂow of 1.0 ml/min and with a linear gradient of 5–50% B in 20 min, with a retention time of 10.3 and 13.6 min, respectively. Buffer A: 10 mM TEAAc (pH 7.1); B: 50% acetonitrile in 10 mM TEAAc (pH 7.1).

To determine the extinction coefficient of 4-Se-uridine residue (^Se^U) by comparing with the native nucleotide, we synthesized and purified the ^Se^UMP and 5′-^Se^UU-3′. Their HPLC profiles are presented in Figure 3. The HPLC assistance, which removes and minimizes the interference of impurities, allows accurate measurement of the extinction coefficients (43). Our experimental results indicate that ^Se^U residue absorbs at both 260 and 370 nm (Figure 3A). The absorption ratio at these two wavelengths is 5.71, calculated on the basis of the HPLC peak areas. As the extinction coefficient is proportional to the absorption, Equation (1) is deduced. In addition, from the HPLC profile (Figure 3B) of 5′-^Se^UU-3′, the ratio between the absorption at 260 nm (contributed by both native U and ^Se^U) and 370 nm (only by ^Se^U) is determined as 0.920. Thus, Equation (2) is deduced. As the extinction coefficient of native U at 260 nm (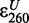 = 9.66 × 10^3 ^M^−^^1^cm^−^^1^) is known (45), we calculated the extinction coefficient of ^Se^U at 370 nm (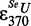) and 260 nm (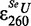) are 13.0 and 2.28 × 10^3 ^M^−^^1^cm^−^^1^, respectively.
(1)
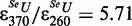

(2)
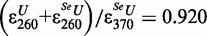

### Thermodenaturation study

**Figure 4.**
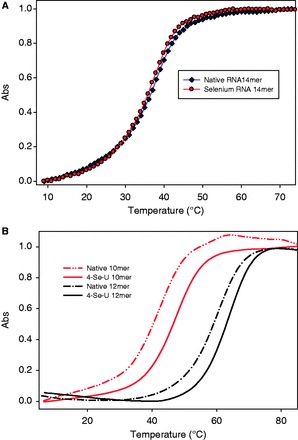
(**A**) Normalized Tm curve of Se-RNA (5′-UUA-^Se^U-AUAUAUAUAA-3′)_2_, compared with the corresponding native RNA. The Se-RNA (circle line): Tm = 37.3 ± 0.5°C; the native (diamond line): Tm = 38.0 ± 0.3°C. (**B**) Normalized Tm profiles of Se-RNA 10mer (5′-rU-^Se^U-AGCUAGCU-3′)_2_ and 12mer (5′-U-^Se^U-CGCGAUCGCG-3′)_2_, compared with their corresponding natives. The native RNA-10mer (gray dash-dot line): Tm = 42.2 ± 0.2°C; the Se-RNA 10mer (gray solid line): Tm = 47.1 ± 0.3°C; the native RNA 12mer (black dash-dot line): Tm = 59.4 ± 0.3°C; the Se-RNA 12mer (black solid line): Tm = 63.2 ± 0.3°C.

The rationales of using a Se atom to probe the U-A and U•U base pairs are that selenium, a large-size atom, can probably strengthen the stacking interaction and is a poorer hydrogen-bond acceptor (30,32,33) that can likely weaken the hydrogen-bond (H-bond) interaction. The polarizable and large Se atom with delocalizable electrons can increase the stacking interaction by narrowing the gap between the stacked nucleobases, which is observed in our crystal structure presented in this work. Furthermore, the increase of the stacking interaction by this Se atomic probe is consistent with the computational study of the Se-modified thymidine in DNA duplex (46). Thus, the Se-atom probe that alters the stacking and H-bonding interactions may provide novel insights into the base pairs. To investigate the RNA duplex recognition and stability, we carried out the UV-melting study with RNAs containing the 4-Se-uracil in duplexes or in duplex junctions (or overhang regions). Typical curves of Se-RNA melting-temperatures (Tm) are showed in Figure 4, and all the Tm data are summarized in Table 2, compared with the corresponding native RNA duplexes. When the Se-atom probe is introduced to the uracil in RNA duplexes, no significant Tm differences between the native and Se-modified duplexes were observed (entry 1–8 in Table 2), and the free energy (ΔG) differences with the corresponding natives were almost zero. This suggests that the Se-atom probe in RNA duplex regions may not cause significant perturbation in duplex stability. As selenium is a poor H-bond acceptor, it is anticipated that the Se-mediated H-bond in the U-A pair is weak. The zero (or very small) free energy difference between the native and Se-modified RNA duplexes also indicates that the stability increase via the stronger stacking compensates the stability decrease caused by the weaker H-bonding. This observation reveals that the modified U-A base-pair can maintain a fine balance between the stacking and H-bonding interactions.

**Table 2.** UV-melting temperatures of ^Se^U-RNAs

Entry	Modified region	RNA sequences	Tm (°C)	ΔTm (°C)
1		(5′-rUUAUAUAUAUAUAA-3′)_2_	38.0 ± 0.3	
2	Duplex	(5′-rUUA-^Se^U-AUAUAUAUAA-3′)_2_	37.3 ± 0.5	−0.7
3		(5′-rGGUAUUGCGGUACC-3′)_2_	45.0 ± 0.4	
4	Duplex	(5′-rGG-^Se^U-AUUGCGGUACC-3′)_2_	44.2 ± 0.3	−0.8
5		(5′-rCGCGAAUUCGCG-3′)_2_	39.4 ± 0.4	
6	Duplex	(5′-rCGCGAAU-^Se^U-CGCG-3′)_2_	39.0 ± 0.3	−0.4
7		5′-AUCACCUCCUUA-3′	43.2 ± 0.3	
		3′-UAGUGGAGGAAU-5′		
8	Duplex	5′-A-^Se^U-CACCUCCUUA-3′	42.8 ± 0.4	−0.4
		3′-U–A–GUGGAGGAAU-5′		
9		(5′-UUAGCUAGCU-3′)_2_	42.2 ± 0.2	
10	Duplex junction	(5′-U-^Se^U-AGCUAGCU-3′)_2_	47.1 ± 0.3	+4.9
11		(5′-UUCGCGAUCGCG-3′)_2_	59.4 ± 0.3	
12	Duplex junction	(5′-U-^Se^U-CGCGAUCGCG-3′)_2_	63.2 ± 0.3	+3.8
13		5′-UUCAUGUGACC-3′	48.2 ± 0.3	
		3′—–GUACACUGGUU-5′		
14	Duplex junction	5′-U-^Se^U-CAUGUGACC-3′	49.9 ± 0.4	+1.7
		3′——–GUACACUGGUU-5′		

It is reported that a U•U pair is less stable comparing with a U-G or C-A mispair in a RNA duplex (33,47). In RNA duplex junctions and loops, however, the two consecutive U•U pairs are more stable than the two consecutive A-A pairs (48). Thus, the Se-atom probe is used to investigate the non-canonical U•U pair, and we chose and modified the RNAs forming RNA duplex and UU junction (Table 2). The UV-thermal denaturation study was carried out, and the melting-temperatures (Tm) of the Se-RNAs and their corresponding natives are summarized in Table 2 (entry 9–14). Excitingly, when the atomic probe is introduced to the RNA duplex junctions, the melting temperatures increased by 1.5–2.4^°^C per Se-modification of these RNA duplexes. Consistently, the free energy (ΔG) calculation indicates that each Se atom contributed additional stabilization (0.4–0.8 kcal/mol) to the stability of the RNA duplexes. This increased RNA duplex stability is attributed to the increased stacking interaction of ^Se^U on the duplex ends; the support from the high-resolution structure data is presented later. Via the Se-atom probe, the UV-melting study of the duplex RNAs containing the UU junction indicates that the uracil stacking contributes significantly to RNA duplex stability.

### Crystallization, diffraction data collection and crystal structure determination

**Figure 5.**
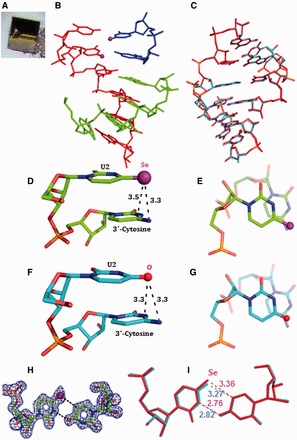
The yellow crystal and structures of the 4-Se-U RNA hexamer, (5′-U-^Se^U-CGCG-3′)_2_. The purple and red balls represent Se and O atoms, respectively. (**A**) The picture of the yellow Se-RNA crystal (0.1 × 0.1 × 0.1 mm). (**B**) Structure of the Se-RNA duplex containing the Se-RNA hexamer (in red), the base-paired CGCG (in green) and the U•U-paired U-^Se^U (in blue). (**C**) Superimposition of the Se-modified structure (in red; PDB ID: 3HGA; 1.30 Å resolution) and the native structure (in cyan; PDB ID: 1OSU; 1.40 Å resolution), the rmsd value is 0.09 Å. (**D**) Se-modified U2 stacks on its 3′-cytosine; the distance between the Se atom and exo-N4 of 3′-cytosine is 3.3 Å; the distance between the Se atom and C4 of 3′-cytosine is 3.5 Å. (**E**) is the top view of (D). (**F**) Native U2 stacks on its 3′-cytosine; the distance between the O atom and exo-N4 of 3′-cytosine is 3.3 Å; the distance between the O atom and C4 of 3′-cytosine is 3.3 Å. (**G**) is the top view of (F). (**H**) Electron density map (2Fo-Fc) and model of the ^Se^U•U pair at the level of 1.0 σ. (**I**) Superimposition of ^Se^U•U pair (in red) with native U•U pair (in cyan); the H-bond lengths are indicated individually.

**Figure 6.**
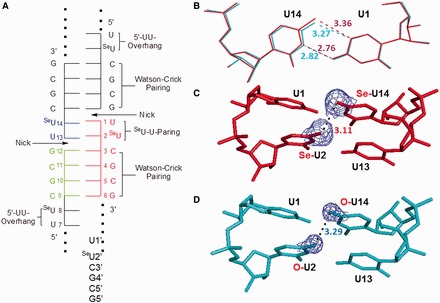
Schematic diagram and local structures of the native and modified U•U pairs in overhang regions (or duplex junctions). (**A**) Schematic diagram of the RNA duplex with five strands, the nicks, ^Se^U•U pairs and normal Watson–Crick C-G pairs. (**B**) Superimposition comparison of ^Se^U14•U1 (in red) with native U14•U1 pair (in cyan); the numbers represent the H-bond lengths (Å). (**C**) The stacking of two ^Se^U•U pairs with the distance (3.11 Å) between the two neighbor Se atoms in the modified U14 and U2. (**D**) The stacking of two native U•U pairs with the distance (3.29 Å) between the two neighbor O atoms in native U14 and U2. The 2Fo-Fc maps of Se-4 and O-4 are showed.

To investigate the Se-nucleobase modification and its structural property, we have crystallized two Se-RNA sequences [hexamer (5′-rU-^Se^U-CGCG-3′)_2_ with overhangs and octamer (5′-rGUG-^Se^U-ACAC-3′)_2_ with a perfect duplex]. Crystals of both Se-RNA sequences were formed in 2–5 days at room temperature (25^°^C) with the Hampton nucleic acid mini-screen kit (total 24 buffers with broad conditions). Excitingly, all crystals of both Se-RNAs had strong yellow or dark yellow color because of the selenium modification (Figures 5 and 6). The Se-RNA hexamer formed crystals in 22 of 24 buffers using the kit, whereas the corresponding native RNA formed crystals only in 4 of 24 buffers (in 3 weeks) using the kit. Most of these Se-RNA crystals (one example shown in Figure 5) diffracted very well, up to 1.3 Å resolution (the orthorhombic space group, C222_1_). Similarly, the Se-RNA octamer formed crystals in 22 of 24 buffers using the same kit, and these crystals (examples shown in Figure 6) could diffract up to 2.5 Å resolution (the rhombohedral space group, R32). In contrast, the corresponding native (5′-rGUGUACAC-3′)_2_ did not crystallize under any conditions over several weeks, which is consistent with the literature (49). The native octamer (5′-rGUGUACAC-3′)_2_ is difficult to crystalize, and its structure has not been reported in literature. Finally, several high-quality crystals from these two Se-RNAs were mounted and cryo-protected for the diffraction data collection. The structures were determined using the best data sets and diffractions collected from the crystals grown in buffer No.10 [10% MPD, 40 mM Na Cacodylate (pH 6.0), 12 mM Spermine tetra-HCl, 12 mM NaCl and 80 mM KCl] for the Se-hexamer and No.12 [10% MPD, 40 mM Na Cacodylate (pH 6.0), 12 mM Spermine tetra-HCl, 80 mM KCl and 20 mM BaCl_2_] for the Se-octamer. The statistic data of the structural analysis are summarized in Table 3, and the determined Se-RNA structures are presented in Figures 5 and 6.

**Table 3.** Diffraction data collection and refinement statistics of the Se-RNA structures

Structure (PDB ID)	U-^Se^U-CGCG (3HGA)	GUG-^Se^U-ACAC (4IQS)
Data collection	Se-Hexamer	Se-Octamer
Space group	C222_1_	R3_2_
Cell dimensions: *a*,*b*,*c* (Å)	30.255, 34.079, 28.931	47.006, 47.006, 354.105
α, β, γ (°)	90, 90, 90	90, 90, 120
Resolution range, Å (last shell)	50.00–1.30 (1.32–1.30)	50.0–2.60 (2.69–2.60)
Unique reflections	3773 (162)	8915 (846)
Completeness%	95.9 (90.0)	99.2 (95.8)
R_merge_%	4.5 (26.1)	5.3 (35.8)
I/σ(I)	40.5 (1.2)	35.9 (1.0)
Redundancy	11.7 (4.2)	10.0 (6.1)
Refinement		
Resolution range, Å	22.62–1.30	31.73.0–2.60
R_work_%	18.9	19.4
R_free_%	22.5	25.8
Number of reflections	3586	4776
Number of atoms		
Nucleic acid (single)	157	1002
Heavy atoms and ion	1 Se	6 Se
Water	42	0
R.m.s. deviations		
Bond length, Å	0.005	0.008
Bond angle, °	0.931	1.846

R_merge_ = 
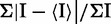

#### Structures of 4-Se-derivatized RNAs

The structure of the Se-RNA hexamer (Figure 5) revealed formation of the right-handed Watson–Crick duplex (Supplementary Table S1) and Hoogsteen base pairs. The structures determined via SAD and molecular replacement approaches are identical. The Se-modified structure (PDB ID: 3HGA; 1.30 Å resolution) and the corresponding native structure (PDB ID: 1OSU; 1.40 Å resolution) (50) are virtually identical as well. They can superimpose on each other perfectly well (Figure 5C) with the RMSD as 0.09 Å, indicating the fine structure isomorphism. Moreover, the electron delocalization of the large Se atom on the uracil may facilitate the nucleobase stacking interaction, also supported by the computational study of the Se-modified nucleobase (46). Furthermore, Se atom is 0.43 Å larger than O, and the distances between U2 4-exo-Se and the 3′-cytosine atoms (N3, exo-N4, C4 and C5) are similar to the corresponding native distances between U2 4-exo-O and the 3′-cytosine atoms (Figure 5D and E); the distances between the 4-Se or 4-O atom and the 3′-C atoms are also displayed. Thus, the comparison of the Se-modified and native structures (Figure 5D-I) suggests that the Se-nucleobase may better stack on the 3′-cytosine than the native nucleobase. The stronger stacking interaction can rigidify the local conformation and strengthen the RNA duplexes, which are consistent with the stronger duplex stability in the presence of the UU overhang (or duplex junction; Table 2). These results are also consistent with the faster crystal growth after the selenium modification. Similar to the corresponding native structure (50), two ^Se^U•U pairs (Hoogsteen pair) have been observed in the Se-RNA (Figure 5F and G). In the Se-modified and native structures, both ^Se^U•U and U•U pairs participate in formation of a pseudo-fiber and long duplex through the overhang Hoogsteen-base pairs. The 5′-UU sequence allows the RNAs (both the Se-modified and native ones) infinitely stacking and elongating along the 2_1_ screw axis in the crystals with nicks on the 5′-end of each 5′-U(^Se^U). This 5′-U-^Se^U sequence forms the two symmetrical ^Se^U•U base pairs, which is virtually identical to the native U•U pair (Figure 5G). Namely, this junction sequence forms the two symmetrical ^Se^U•U base pairs, which glue the RNA duplexes together in a head-to-tail linear fashion.

The results of our crystal structure study are consistent with the UV-melting study. The 5′-UU of one RNA molecule (e.g. the red one in Figure 6A) forms two U•U pairs with the second RNA molecule (the blue one), whereas its consecutive CGCG sequence forms regular Watson–Crick base pairs with the third RNA molecule (the green one). As showed in Figure 5F, the ^Se^U•U pair displays a conventional hydrogen bond between O4 of the native uracil (U1) and N3 of the Se-uracil (U14) and an unusual C-H**^…^**Se hydrogen bond between C5 of native U and Se4 of Se-U, through the Hoogsteen edge of native U and the Watson–Crick edge of Se-U. These interactions result in a *trans*-Hoogsteen U•U pair (Figure 5F). Compared with the native structure, the substitution of the uridine 4-oxygen with a selenium atom does not change the structure significantly (Figure 5C), suggesting that the Hoogsteen U•U pair has space available at 4-position of the Watson–Crick edge. A slight shift (0.09 Å) on the Se-modified nucleobase is observed (Figure 6B). The Hoogsteen C-H**^…^**Se (or O) hydrogen bond (bond length: 3.36 Å in the Se case), between C5 of native U and Se4 of Se-U (the corresponding native H-bond: 3.27 Å; Figure 6B), is still retained. Because selenium atom (1.16 Å in atomic radius) is 0.43 Å larger than oxygen (0.73 Å in atomic radius), it is surprising to find the nucleobase shift only by 0.09 Å to accommodate the big selenium atom, confirming that the native hydrogen bond (O4^…^H-C5) of the Hoogsteen pair is weak. Thus, the large Se atom probe indicates that the Hoogsteen H-bond is less important in the U•U pairing. This also suggests that the *trans*-Hoogsteen pair can tolerate a larger substitution and that the Hoogsteen pair is not rigid, which gives the duplex junction sufficient flexibility. Moreover, it is counterintuitive that the distance (3.11 Å) between these two big neighboring 4-Se atoms (Figure 6C) is even smaller (by 0.18 Å) than the native distance (3.29 Å) between these two small O atoms (Figure 6D), implying the enhanced stacking interactions between these two U•U pairs. Using electron-rich selenium as the atomic probe, our structural result suggests the strong electron delocalization and stacking interaction between these two U•U pairs. The structure study provides new insights into the Hoogsteen U•U pair and the uracil-mediated interactions in ncRNAs.

**Figure 7.**
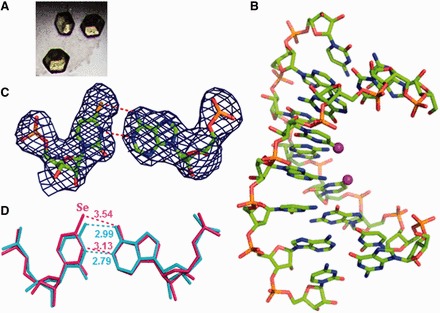
The yellow crystals and structures of the 4-Se-U RNA octamer, (5′-GUG-^Se^U-ACAC-3′)_2_. The Se atoms are labeled as purple balls. (**A**) Crystal image. (**B**) The Se-RNA duplex structure (PDB ID: 4IQS; 2.75 Å resolution). (**C**) Electron density map (2Fo-Fc) and model of the ^Se^U-A pair at the level of 1.0 σ. (**D**) Superimposition of ^Se^U-A pair (in pink) with native U2-A15 pair (in cyan); the H-bond lengths are indicated individually.

The Se-octamer structure (Figure 7), where the two Se atoms point to the major groove, reveals formation of the ^Se^U-A pair and the typical right-handed A-form duplex by the Se-RNA (Supplementary Table S2). Moreover, we have superimposed the structures of ^Se^U-A (or ^Se^U4-A13 pair) and U2-A15 pair (Figure 7D), as the corresponding native structure is not available (from literature or us) for direct comparison. This comparison of the base pair structures has demonstrated that the Se-modified and native U-A pairs are similar. The major difference is the slight shift of the ^Se^U nucleobase to accommodate the large selenium atom, revealing the flexibility of RNA duplex structure. The distance between ^Se^U4 exo-Se4 and A13 exo-N6 is 3.54 Å, which was increased from the original 2.99 Å. Considering that the atomic size of Se is 0.43 Å larger than that of O and that a typical H-bond length is 2.8–3.2 Å, this distance (3.54 Å) suggests a weak hydrogen bond after the Se-modification. On the other hand, the polarizable and large Se atom with delocalizable electrons may facilitate the base stacking interaction, supported by the narrower base-pair gap and the computational study of the Se-nucleobase-modified DNA (46). Using the Se atom probe, we found that the increased stacking interaction can compensate the loss of the H-bond interaction, which is consistent with the virtually identical duplex stability after the Se-modification (Table 2). Moreover, most of the 2′-hydroxyl groups are involved in the H-bonding interactions with its 3′-sugar ring oxygen (O4′) or 3′-phosphate oxygen, which restrains the conformations of the sugar-phosphate backbone, thereby facilitating the intramolecular interaction and reducing molecular dynamics. The Se-RNA crystallization is consistent with the Se-enhanced base stacking and conformation rigidification. In the crystal lattice, the duplexes are stacked on the top of each other in a head-to-tail fashion and three Se-RNA duplexes present in an asymmetric unit, where the three duplexes are virtually identical (r.m.s < 0.1 Å). Chain A and B are showed in Figure 7.

Furthermore, X-ray crystallography is one of the most powerful methodologies for structure and function studies of RNAs and their complexes with ligands, including protein-RNA complexes and RNA-small molecule complexes, at the atomic resolution. However, owing to the difficulties in crystallization and phasing (phase determination or phase problem), progress in RNA crystallography is limited, especially in the ncRNA structure study. Inspired by the protein Se-derivatization, multi-wavelength anomalous dispersion phasing and SAD phasing (51–55), our laboratory has pioneered SeNA (36,37), which has great potential as a general strategy for RNA X-ray crystallography (37). This research work on the synthesis and structure studies of the 4-Se-uridine RNAs has further demonstrated that the selenium modification is a useful approach for structural biology, as the Se-functionalization can facilitate phase determination, crystallization, RNA color and atomic probing.

## CONCLUSION

To probe uracil-mediated interactions and base-pairs with a single selenium atom, we have synthesized the 4-Se-uridine phosphoramidite and Se-RNAs. Our thermostability and structure studies indicate that the modified and native structures are virtually identical, that the H-bonding decrease in U-A pair can be compensated by the base-stacking increase, and that the uracil stacking in duplex junction may increase duplex thermostability. We also found that the stacking interaction of the two *trans*-Hoogsteen U•U pairs is the main contributor to the duplex junction stability, whereas the Hoogsteen H-bond is weak. Moreover, the accommodation of larger Se atoms in uracil by both U-A and U•U pairs implies the RNA flexibility. Using the Se atom probe, our studies confirm that uracil is capable of interacting in multiple modes, thereby diversifying U•U and U-A pairs in structure and function. Our thermodynamic and structural studies have also demonstrated that this Se-modification can facilitate the nucleobase stacking interaction and potential crystal growth without significant perturbation. Furthermore, this Se-modification generates color RNA for the first time by single atom replacement, and it shifts the uridine UV spectrum over 100 nm (^Se^U λ_max_: 370 nm; **ε:** 1.30 × 10^4^ M^−^^1^cm^−^^1^). This color property is useful for RNA-protein co-crystallization, RNA visualization, detection and spectroscopic study. This work provides a new strategy for crystallization, phasing, structure and function studies of ncRNAs and protein-RNA complexes.

## ACCESSION NUMBERS

3HGA, 4IQS

## SUPPLEMENTARY DATA

Supplementary Data are available at NAR Online.

Supplementary Data
