# Supplementary material for: Structural insights of non-canonical U•U pair and Hoogsteen interaction probed with Se atom
Source: Nucleic Acids Res. 2013 Sep 5;41(22):10476–87. doi: 10.1093/nar/gkt799 (PMC3905866; doi:10.1093/nar/gkt799)
Supplement: Supplementary Data [file supp_41_22_10476__index.html]

Structural insights of non-canonical U•U pair and Hoogsteen interaction probed with Se atom — Structural insights of non-canonical U•U pair and Hoogsteen interaction probed with Se atom — Supplementary Data 

# Structural insights of non-canonical U•U pair and Hoogsteen interaction probed with Se atom

## Supplementary Data

files

**Files in this Data Supplement:**

- Supplementary Data - doc file
