# Supplementary material for: Structural insights of non-canonical U•U pair and Hoogsteen interaction probed with Se atom
Source: Nucleic Acids Res. 2013 Sep 5;41(22):10476–87. doi: 10.1093/nar/gkt799 (PMC3905866; doi:10.1093/nar/gkt799)
Supplement: Supplementary Data [file supp_gkt799_nar-01399-r-2013-File002.doc]

Supporting Information

Structural Insights of Non-canonical U/U Pair and Hoogsteen Interaction Probed with Se Atom

Jia Sheng^1^, Jianhua Gan^1^, Alexie Soares^2^, Jozef Salon^1^ and Zhen Huang^1^*

1. Department of Chemistry, Georgia State University, Atlanta, GA, 30303

2. Department of Biology, Brookhaven National Laboratory, Upton, NY, 11973

[huang@gsu.edu](mailto:Huang@gsu.edu)

**Table of Contents**

General Experimental Section………………..……………...………..….S1

Mass and NMR and HR-MS Spectra of Compound **2** and **3**…………….S2-5

HPLC Analysis, Purification and Characterization………………..…......S5-6

Helical Parameter Tables …………………………………….…………..S6

**General procedures**

Anhydrous and air-sensitive solvents and reagents were used and stored in between uses in a Vacuum Atmospheres Company (VAC) M040-2 glove box that was pressurized with nitrogen boil-off gas from a liquid nitrogen tank or in a VAC CS-40 glove box freezer at –20 °C. All starting materials for anhydrous reactions were dried prior to use on a vacuum line (1 - 4 × 10^–4^ torr). Reactions were monitored with glass-backed TLC plates pre-coated with silica gel 60 F254 (EMD Chemicals). Flash column chromatography was carried out using Fluka silica gel (60Å pore, 230-400 mesh) that was packed in glass columns and pressurized with nitrogen. NMR Spectra were recorded on a Varian Unity +300 or Brucker Avance 400 spectrometer. Chemical shifts for ^1^H NMR were referenced relative to tetramethylsilane (0.00 ppm), CDCl_3_ (7.24 ppm) or DMSO (2.50 ppm).  Chemical shifts for ^13^C NMR were referenced relative to CDCl_3_ (77.23 ppm) or DMSO (39.50 ppm). ^13^C NMR signals were assigned using 13C-APT technique. High resolution MS (HR-MS) spectra were obtained with electrospray ionization (ESI) on a Q-TOFTM Waters Micromass at Georgia State University.


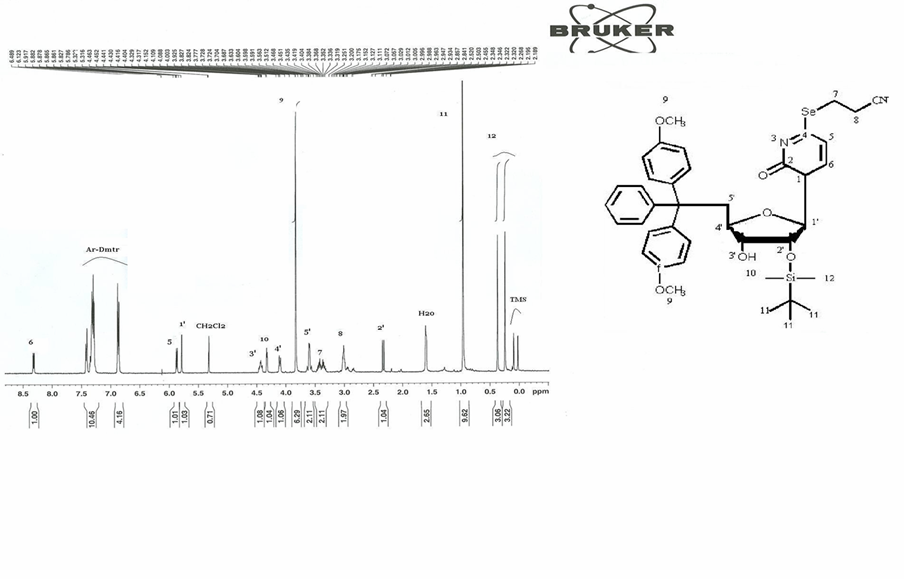


**Figure S1:** ^1^H-NMR spectrum of ^Se^U-intermediate **2**.

**
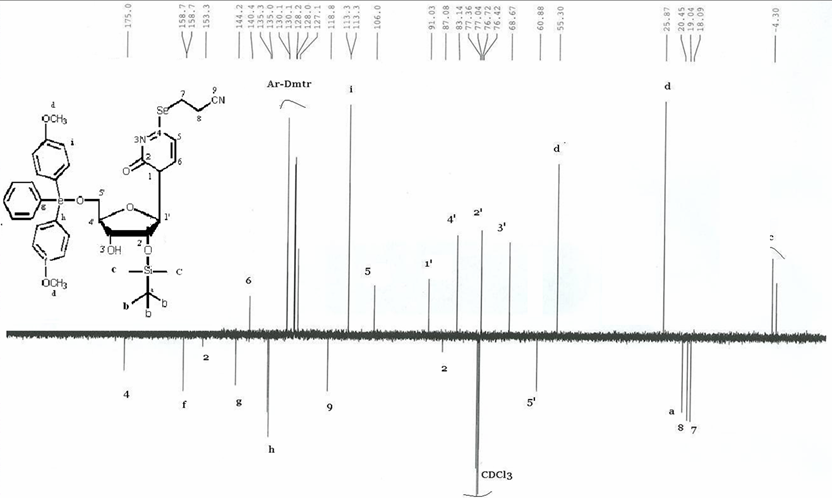
**

**Figure S2**. ^13^C-NMR spectrum of ^Se^U-intermediate **2**.


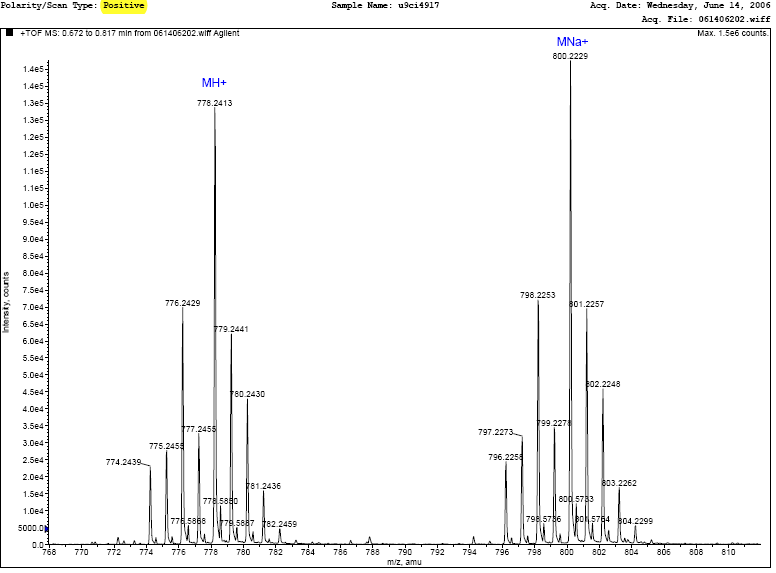


**Figure S3** HRMS (ESI) spectrum of ^Se^U-intermediate **2**. Molecular formula, C_39_H_49_N_3_O_7_SeSi; [M+H^+^]^+^: 778.2413 (calc.778.2426).


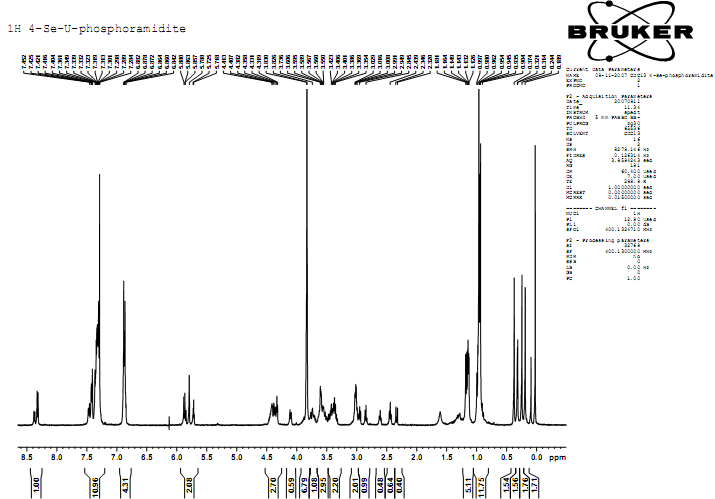


**Figure S4**. ^1^H-NMR spectrum of ^Se^U -phosphoramidite **3**.

***
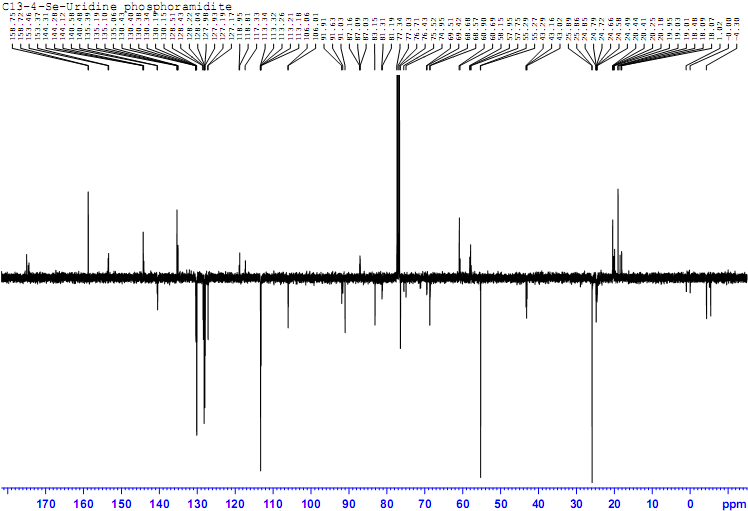
***

**Figure S5.** ^13^C-NMR spectrum of ^Se^U-phosphoramidite **3**.

**
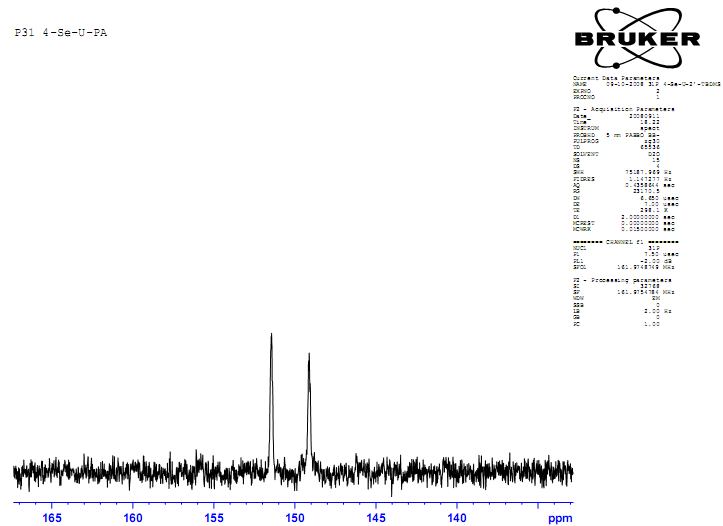
**

**Figure S6**: ^31^P-NMR spectrum of ^Se^U-phosphoramidite **3**.

**Figure S7**: HRMS (ESI) spectrum of ^Se^U phosphoramidite **3**. Molecular formula, C_48_H_64_N_5_O_8_PSeSi; [M+H^+^]^+^: 978.3479 (calc. 978.3505).

**HPLC analysis, purification and characterization**

**Figure S8.** RP-HPLC analysis of the crude ^Se^U-RNA. The RNA sample (5'-GUG-^Se^U-ACAC-3'), monitored at 260 nm (profile 1; black) and 370 nm (profile 2; red), was analyzed on a Welchrom XB-C18 column (4.6 x 250 mm, 5 μ) at a ﬂow of 1.0 mL/min and with a linear gradient of 5 to 70% B in 20 min. The Se-RNA retention time is 12.4 min. Buffer A: 10 mM TEAAc (pH 7.1); B: 50% acetonitrile in 10 mM TEAAc (pH 7.10).

**Figure S9.** MALDI-TOF mass spectrum of 4-Se-U-RNA [5’-rGG(^Se^U)AUUGCGGUACC-3’], C_133_H_165_N_52_O_97_P_13_Se, [M+H]^+^: 4526.4 (Calcul. 4526.7).

**Table S1.** Helical parameters of 4-SeU-RNA [U(^Se^U)CGCG]_2_

| Steps | | Shift | Slide | Rise | Tilt | Roll | Twist |
| --- | --- | --- | --- | --- | --- | --- | --- |
| 1 | U/^Se^U | 5.47 | -0.24 | 3.15 | 5.5 | 12.42 | 71.5 |
| 2 | ^Se^U/C | 2.35 | -1.82 | 3.6 | -0.99 | 9.2 | 43.48 |
| 3 | C/G | 0.06 | -1.72 | 3.23 | 0.17 | 17.19 | 30.72 |
| 4 | G/C | 0.63 | -1.04 | 3.18 | 5.91 | 6.26 | 33.23 |
| 5 | C/G | 0.7 | -1.74 | 3.16 | 2.95 | 13.68 | 30.73 |

**Table S2.** Helical parameters of 4-SeU-RNA [GUG(^Se^U)ACAC]_2_

| Steps | | Shift | Slide | Rise | Tilt | Roll | Twist |
| --- | --- | --- | --- | --- | --- | --- | --- |
| 1 | G/U | 0.06 | -1.37 | 3.22 | 2.2 | 6.92 | 35.58 |
| 2 | U/G | 0.64 | -1.68 | 3.12 | 6.42 | 10.03 | 28.45 |
| 3 | G/ ^Se^U | 1.46 | -1.21 | 3.09 | 5.19 | 9.76 | 35.72 |
| 4 | ^Se^U/A | 0.03 | -1.74 | 2.96 | 8.66 | 2.24 | 28.83 |
| 5 | A/C | -0.03 | -1.41 | 3.1 | 8.13 | 6.81 | 26.69 |
| 6 | C/A | 0.26 | -1.59 | 3.41 | -2.67 | 14.52 | 29.09 |
| 7 | A/C | 0.43 | -1.27 | 2.73 | 12.5 | 1.86 | 30.59 |
